# Safety of Biologic Therapy for Inflammatory Bowel Disease in Liver Transplant Recipients

**DOI:** 10.3390/biomedicines14092117

**Published:** 2026-09-19

**Authors:** Tanja Elger, Johanna Loibl, Martina Müller, Sophia Rusch, Lucia Baier-Kleinhenz, Arne Kandulski, Christa Buechler, Hauke Christian Tews

**Affiliations:** 1 Department of Internal Medicine I, Gastroenterology, Hepatology, Endocrinology, Rheumatology, Immunology, and Infectious Diseases, University Hospital Regensburg, 93053 Regensburg, Germany; 2 Department of Surgery, University Hospital Regensburg, 93053 Regensburg, Germany

**Keywords:** inflammatory bowel disease, Crohn’s disease, ulcerative colitis, liver transplant, biologic therapy, primary sclerosing cholangitis, autoimmune hepatitis

## Abstract

**Background/Objectives**: Liver diseases and chronic inflammatory bowel diseases (IBD) are closely associated, particularly primary sclerosing cholangitis (PSC) and ulcerative colitis (UC). In severe cases of PSC, liver transplantation (LT) may be required, just as severe IBD may necessitate biologic therapy. Evidence on combining biologics with post-transplant immunosuppression is limited, particularly regarding hepatotoxicity, infections, and drug–drug interactions. We therefore assessed the safety of biologic therapy for IBD in LT recipients. **Methods**: In this retrospective, single-center analysis, all patients treated at the inflammatory bowel disease and post–liver transplantation outpatient clinics of the University Hospital Regensburg were screened. Patients with both LT and IBD were included in the analysis. Medical records, laboratory parameters, and imaging studies were reviewed. **Conclusions**: A total of 17 liver transplant recipients with IBD were identified. Eleven patients had PSC as the underlying liver disease, while the remaining patients had autoimmune hepatitis (AIH), PSC–AIH overlap syndrome, or cryptogenic liver cirrhosis. Three patients had Crohn’s disease, and 14 had ulcerative colitis. Five patients required biologic therapy because of active IBD. Two patients received two sequential biologic agents, whereas one patient received four different biologics during follow-up. No significant safety signals were observed regarding hepatotoxicity, susceptibility to infections, or interactions with concomitant immunosuppressive therapy, including tacrolimus, mycophenolate mofetil, ciclosporin, and prednisolone. No evidence of clinically relevant safety concerns associated with biologic therapy was identified in liver transplant recipients with IBD. Therefore, biologic therapy combined with conventional post-transplant immunosuppression appears feasible and well tolerated in LT recipients with active IBD; larger prospective studies are needed. However, larger prospective studies are needed to confirm these findings.

## 1. Introduction

IBD, namely Crohn’s disease (CD) and UC, comprises chronic inflammatory disorders of the gastrointestinal tract characterized by abdominal pain, diarrhea and chronic inflammation. Whereas UC affects the colon, Crohn’s disease classically presents as multifocal inflammation with terminal ileitis but can affect the entire gastrointestinal tract [1,2]. Complications include fistulas, stenosis, abscesses, severe malnutrition, impaired quality of life, and an increased risk of colorectal cancer. Although the exact pathogenesis of IBD remains incompletely understood, immunosuppressive therapies have proven to be effective. The therapeutic landscape for IBD has changed dramatically over the past three decades. Whereas treatment was previously limited to corticosteroids and thiopurines for the induction and maintenance of remission, current therapeutic options include anti-TNF agents, integrin antagonists, interleukin (IL)-12/23 and IL-23 inhibitors, as well as small-molecule therapies [3,4].

Liver diseases, particularly PSC, are closely associated with IBD [5]. Approximately 70–80% of patients with PSC have concomitant IBD, with UC accounting for approximately 80% and CD for approximately 15% of PSC-IBD cases [6]. Although numerous immunosuppressive agents, including prednisone, azathioprine, colchicine, tacrolimus, mycophenolate mofetil (MMF), and etanercept, as well as various antibiotics and antifibrotic agents, have been investigated, no immunosuppressive therapy has yet demonstrated efficacy in altering the natural course of PSC. Ursodeoxycholic acid remains the most commonly prescribed medical treatment for PSC [7]. Despite optimized surveillance and supportive treatment, PSC and other chronic liver diseases, such as AIH, may progress to liver fibrosis and cirrhosis, ultimately necessitating liver transplantation [8].

The liver is the most tolerogenic of all transplanted solid organs [9]. Nevertheless, lifelong immunosuppressive therapy is generally required after liver transplantation to prevent graft rejection. Standard immunosuppressive regimens are based on calcineurin inhibitors (CNIs), such as tacrolimus and ciclosporin, in combination with MMF and corticosteroids [10]. Despite this maintenance immunosuppression, some liver transplant recipients with concomitant IBD continue to experience active disease requiring additional biologic therapy to achieve adequate control of intestinal inflammation.

However, the combination of conventional post-transplant immunosuppression with biologic therapy for IBD has not been evaluated in large cohorts and therefore remains an off-label treatment strategy. Consequently, the present study aimed to evaluate the safety of biologic therapy in liver transplant recipients with IBD, with particular emphasis on hepatotoxicity, susceptibility to infections, and potential drug interactions between biologic agents and concomitant immunosuppressive therapy.

## 2. Materials and Methods

This retrospective single-center analysis evaluated the efficacy and safety of biologic therapies for IBD in liver transplant recipients. The primary objective was to evaluate the safety of biologic therapy with regard to hepatotoxicity, infectious complications, and potential drug interactions. Secondary endpoints included changes in liver biochemistry, inflammatory biomarkers, and clinical response during biologic therapy.

All patients attending the outpatient clinics for inflammatory bowel disease and post-liver transplantation follow-up at the Department of Internal Medicine I, University Hospital Regensburg, were screened to identify liver transplant recipients with concomitant inflammatory bowel disease (CD or UC). Detailed case descriptions were compiled for all liver transplant recipients who required biologic therapy for IBD. The data cut-off date was 1 July 2025.

Medical records, laboratory parameters, and imaging studies were reviewed to assess the aforementioned variables. Inflammatory bowel disease was diagnosed according to established endoscopic, histological, and clinical criteria. Statistical analysis was performed using IBM SPSS Statistics version 29.0.0.0 (IBM Corp., Armonk, NY, USA). Paired *t*-test was used to assess statistical significance. A two-sided *p*-value of <0.05 was considered statistically significant. Normality was assessed using Shapiro-Wilk-Test. Statistical comparisons for patients receiving multiple sequential biologic agents were performed at treatment-course level, and measurements describing treatment success were assessed before and during each therapy course. Due to the retrospective design, no standardized washout periods were required, so overlapping treatment periods may have occurred.

## 3. Results

### 3.1. Case Descriptions of Biologic Therapy Recipients

Patients’ medical histories support the conclusion that elevations in liver enzymes and cholestatic markers are generally not attributable to biologic therapy and are therefore presented before the main text.

Case 1: Patient 1 was diagnosed with type 1 AIH in 2003 at the age of 15 years and was initially treated with azathioprine. Despite immunosuppressive therapy, the disease progressed to end-stage liver cirrhosis, and she underwent living-donor liver transplantation in January 2017 using a graft donated by her husband. However, primary graft non-function necessitated re-transplantation with a deceased-donor liver only 10 days later. Owing to recurrent graft failure, a third liver transplantation from a deceased donor was required six days thereafter.

Despite satisfactory graft function following the third transplantation, the postoperative course was complicated by multiple organ failure, dialysis-dependent acute kidney injury, and prolonged mechanical ventilation. In addition, anastomotic leakage of the common bile duct (CBD) required percutaneous transhepatic cholangial drainage (PTCD) for four weeks. The patient recovered and was discharged after a three-month hospital stay. Thereafter, liver function tests remained stable, renal function gradually improved, and maintenance immunosuppression consisted of tacrolimus.

The patient became pregnant in 2017 and 2019 and delivered two healthy sons, althouth multiple endoscopic retrograde cholangiopancreatography (ERCP) procedures with balloon dilatation of an anastomotic CBD stricture were necessary.

In July 2021, she presented with bloody diarrhea, and colonoscopy revealed ulcerative pancolitis with backwash ileitis. Despite treatment with mesalazine and budesonide in addition to tacrolimus-based immunosuppression, adequate disease control could not be achieved. Therefore, vedolizumab therapy was initiated in December 2021. A transient increase in transaminases and cholestatic liver enzymes was considered more likely attributable to recurrent anastomotic CBD stenosis than to vedolizumab-induced hepatotoxicity. Although intermittent diarrhea and hematochezia persisted, her general condition remained good. Because endoscopic assessment demonstrated an insufficient therapeutic response, treatment was switched to ustekinumab in October 2022. Despite subsequent treatment with ustekinumab and later risankizumab, severe pancolitis persisted on endoscopic examination. Consequently, therapy was changed to infliximab in April 2025.

Case 2: Patient 2 was diagnosed with CD in 1998 at the age of 8 years. The disease involved the colon, stomach, esophagus, and oral cavity. Two years later, she was diagnosed with PSC-AIH-overlap syndrome. Initial treatment consisted of ciclosporin and azathioprine, supplemented by several courses of high-dose corticosteroid pulse therapy. Owing to persistent CD activity, treatment was escalated in 2007 to methotrexate (MTX), azathioprine, and corticosteroids.

In 2009, abdominal ultrasound revealed a lesion in liver segment VI, prompting right hemihepatectomy combined with surgical portocaval shunt placement. The postoperative course was complicated by profound leukopenia, most likely induced by metamizole, necessitating temporary discontinuation of immunosuppressive therapy. Following recovery from neutropenia, azathioprine and prednisolone were resumed. Shortly thereafter, however, Crohn’s disease relapsed, while pelvic magnetic resonance imaging (MRI) revealed a perirectal abscess. The patient received intravenous corticosteroids and antibiotic therapy before undergoing colectomy in June 2010.

In 2012, Graves’ disease was diagnosed. Between 2012 and 2013, the patient experienced multiple episodes of cholangitis requiring repeated ERCP and antibiotic treatment. Consequently, she was evaluated for liver transplantation.

In 2014, a progressively enlarging hepatic lesion necessitated central liver resection. Histopathological examination identified the lesion as a hepatocellular adenoma. Only three weeks later, at the age of 24 years, the patient underwent orthotopic liver transplantation. The postoperative course was uneventful, and maintenance immunosuppressive therapy consisted of tacrolimus, MMF and prednisolone. During follow-up, graft function remained stable, and the patient attended regular outpatient follow-up visits. Routine surveillance endoscopy showed only mild inflammation of the remaining rectum, whereas the terminal ileum remained endoscopically unremarkable.

In 2023, CD flared again. Consequently, treatment with vedolizumab was initiated. As stool frequency failed to improve, therapy was switched to ustekinumab. Because no meaningful clinical response was achieved, ustekinumab was discontinued in December 2024. The patient declined further escalation of immunosuppressive therapy, and CD remained clinically active at the end of the observation period.

Case 3: Patient 3 first presented to our hepatology outpatient clinic in 2011 at the age of 26 years after relocating to our region. At that time, he had already been listed for liver transplantation because of end-stage liver cirrhosis secondary to AIH, which had been diagnosed in 1998 at the age of 12 years. As liver disease continued to progress, he underwent orthotopic liver transplantation in early 2015.

Three months after transplantation, the patient developed acute graft rejection and received high-dose corticosteroid pulse therapy. Subsequently, recurrent cholangitis due to an anastomotic biliary stricture developed. ERCP and cholangioscopy demonstrated a caliber mismatch between the donor and recipient bile ducts, necessitating resection of the bile duct with biliodigestive reconstruction.

Despite these interventions and placement of PTCD, the patient continued to experience recurrent episodes of cholangitis complicated by septic shock. Consequently, he was re-listed for liver transplantation and successfully underwent re-transplantation in 2017. Maintenance immunosuppressive therapy consisted of tacrolimus and prednisolone. Thereafter, graft function remained stable, and no further episodes of cholangitis occurred.

In 2019, the patient was diagnosed with UC and started on mesalazine therapy. In 2022, the disease flared, prompting initiation of vedolizumab therapy. Follow-up colonoscopy demonstrated persistent endoscopic inflammation. Consequently, treatment was switched to ustekinumab in June 2024.

Meanwhile, the patient was diagnosed with bullous pemphigoid in 2023. Because he was already receiving systemic immunosuppressive therapy with tacrolimus and prednisolone, the dermatological manifestations were managed with topical corticosteroids alone.

Case 4: Patient 4 was diagnosed with ulcerative colitis in 1995 at the age of 35 years. Initial treatment with 5-aminosalicylic acid (5-ASA) achieved good disease control. One year later, PSC was diagnosed, and treatment with ursodeoxycholic acid was initiated.

Over the following years, the patient developed a clinically relevant biliary stricture at the hepatic hilum, requiring multiple ERCP procedures with biliary stent placement. Consequently, he was evaluated for liver transplantation in 2005 and underwent orthotopic liver transplantation with biliodigestive reconstruction in 2008.

Four weeks after transplantation, the postoperative course was complicated by an enterocutaneous fistula originating from the small intestine at the biliodigestive anastomosis, which required surgical revision. Maintenance immunosuppressive therapy consisted of tacrolimus and prednisolone. Thereafter, both PSC and UC remained clinically stable until 2014, when magnetic resonance cholangiopancreatography (MRCP) and laboratory finding indicated recurrent cholangitis. Liver biopsy subsequently demonstrated active fibrosing pericholangitis with incomplete cirrhosis, consistent with recurrent PSC. Because of recurrent episodes of cholangitis, the patient was re-evaluated for liver transplantation in 2016 and underwent re-transplantation in January 2018. Following transplantation, PSC remained well controlled. Maintenance immunosuppression consisted of MMF and tacrolimus.

Despite baseline immunosuppression and treatment with mesalazine, ulcerative colitis became increasingly active, requiring repeated courses of corticosteroid pulse therapy and prompting initiation of vedolizumab therapy. The patient subsequently achieved sustained clinical remission.

In 2024, he developed a symmetrical pretibial erythematous papular rash, clinically consistent with leukocytoclastic vasculitis. The patient reported worsening of the skin lesions after each vedolizumab infusion. However, the rash resolved completely with topical corticosteroid therapy. Given the excellent clinical response of UC, vedolizumab was continued, and no further escalation of dermatological treatment was required.

Case 5: Patient 5 underwent orthotopic liver transplantation for autoimmune hepatitis in 2000 in the Czech Republic. In 2019 he presented to our liver transplant outpatient clinic and his maintenance immunosuppressive therapy consisted of ciclosporin and prednisolone. The patient’s medical history was notable for urothelial carcinoma of the bladder, treated surgically in 2016, and superficial spreading melanoma, which had been completely resected in 2017. Routine oncological follow-up demonstrated no evidence of disease recurrence.

In 2023, UC was diagnosed in colonoscopy, as the patient reported frequent bloody diarrhea. Treatment with mesalazine and budesonide resulted in initial clinical improvement, allowing continuation of mesalazine as maintenance therapy. However, ulcerative colitis relapsed in 2024, prompting initiation of vedolizumab. At the end of follow-up, ulcerative colitis remained well controlled under vedolizumab therapy.

### 3.2. Statistical Analysis

#### 3.2.1. Patient Characteristics

A total of 17 liver transplant recipients with concomitant IBD were identified. Of these, 12 (70.6%) were male and 5 (29.4%) were female (Table 1). The mean age at data collection was 50.4 years (range, 34–67 years). Five patients (29.4%) required biologic therapy because of active IBD despite maintenance immunosuppression following liver transplantation.

The most common underlying liver disease was PSC, affecting 11 of 17 patients (64.7%). Interestingly, among patients requiring biologic therapy, AIH was more common than PSC, accounting for three of five patients (60%).

The majority of patients had UC (82.4%), whereas CD was present in three patients (17.6%). All patients underwent liver transplantation. Four patients (23.5%) required re-transplantation, and one patient underwent three consecutive liver transplantations.

Maintenance immunosuppression after liver transplantation mainly consisted of tacrolimus, MMF, and corticosteroids, both in patients receiving biologic therapy and those managed without biologics.

Vedolizumab was the first-line biologic agent in all five patients requiring biologic therapy with 5 treated patients in total. In cases of insufficient treatment response, therapy was sequentially escalated to ustekinumab (*n* = 3), risankizumab (*n* = 1), and infliximab (*n* = 1). The total number of treatment months for each biologic were 87 months for Vedolizumab, 47 Months for Ustekinumab, 5 months for Risankizumab and 3 months for infliximab and can be found in Table 2.

#### 3.2.2. Liver Function Tests

**Figure 1:**
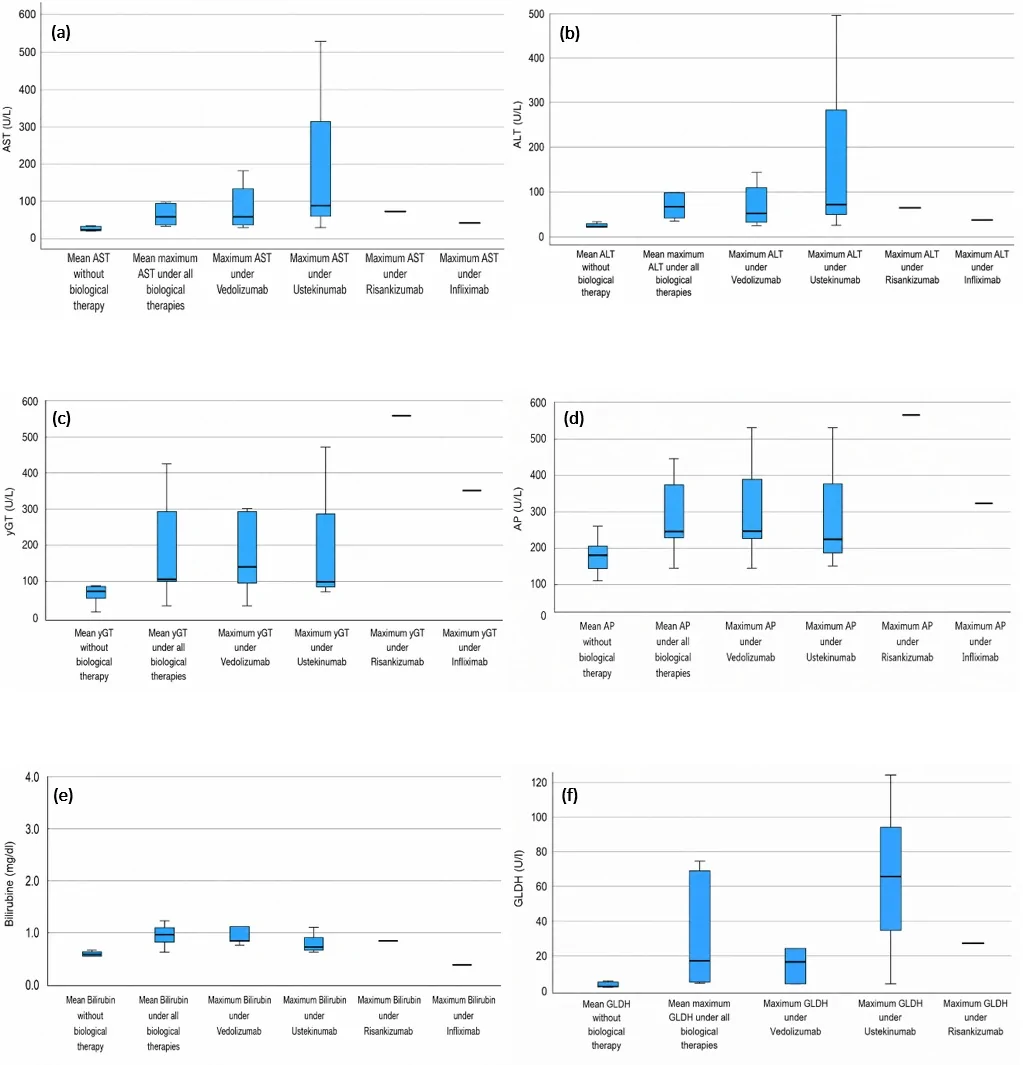
Liver function tests of patients without and during biologic therapy. (**a**) AST (U/L); (**b**) ALT (U/L); (**c**) gamma-glutamyltransferase (γGT) (U/L); (**d**) alkaline phosphatase (AP) (U/L); (**e**) Bilirubin (mg/dL); (**f**) glutamate dehydrogenase (GLDH) (U/L).

Alanine aminotransferase (AST) levels increased during most biologic treatment courses (Figure 1a). However, this increase did not reach statistical significance (*p* = 0.11). In nearly all cases, AST values subsequently decreased spontaneously and remained below three times the upper limit of normal (ULN). A comparable pattern was observed for alanine aminotransferase (ALT) (Figure 1b).

γGT, AP, and bilirubin increased significantly during biologic therapy (Figure 1c–e). The highest γGT and AP values were observed during risankizumab therapy. GLDH levels also increased during treatment (Figure 1f). Although the small sample size and the limited number of patients per individual agent preclude definitive conclusions, it seems that none of these laboratory changes translated into clinically overt hepatotoxicity.

#### 3.2.3. Adverse Events

Only a small number of adverse events were observed during biologic therapy (Table 3). During vedolizumab therapy, one patient developed an uncomplicated upper respiratory tract infection with flu-like symptoms, whereas another patient experienced atypical pneumonia. In the latter patient, cytomegalie virus (CMV) colitis occurred approximately one year later. One patient developed leukocytoclastic vasculitis, which was considered possibly related to vedolizumab therapy. During ustekinumab therapy, hair loss in one patient was the only recorded adverse event.

#### 3.2.4. Inflammatory Markers

**Figure 2:**
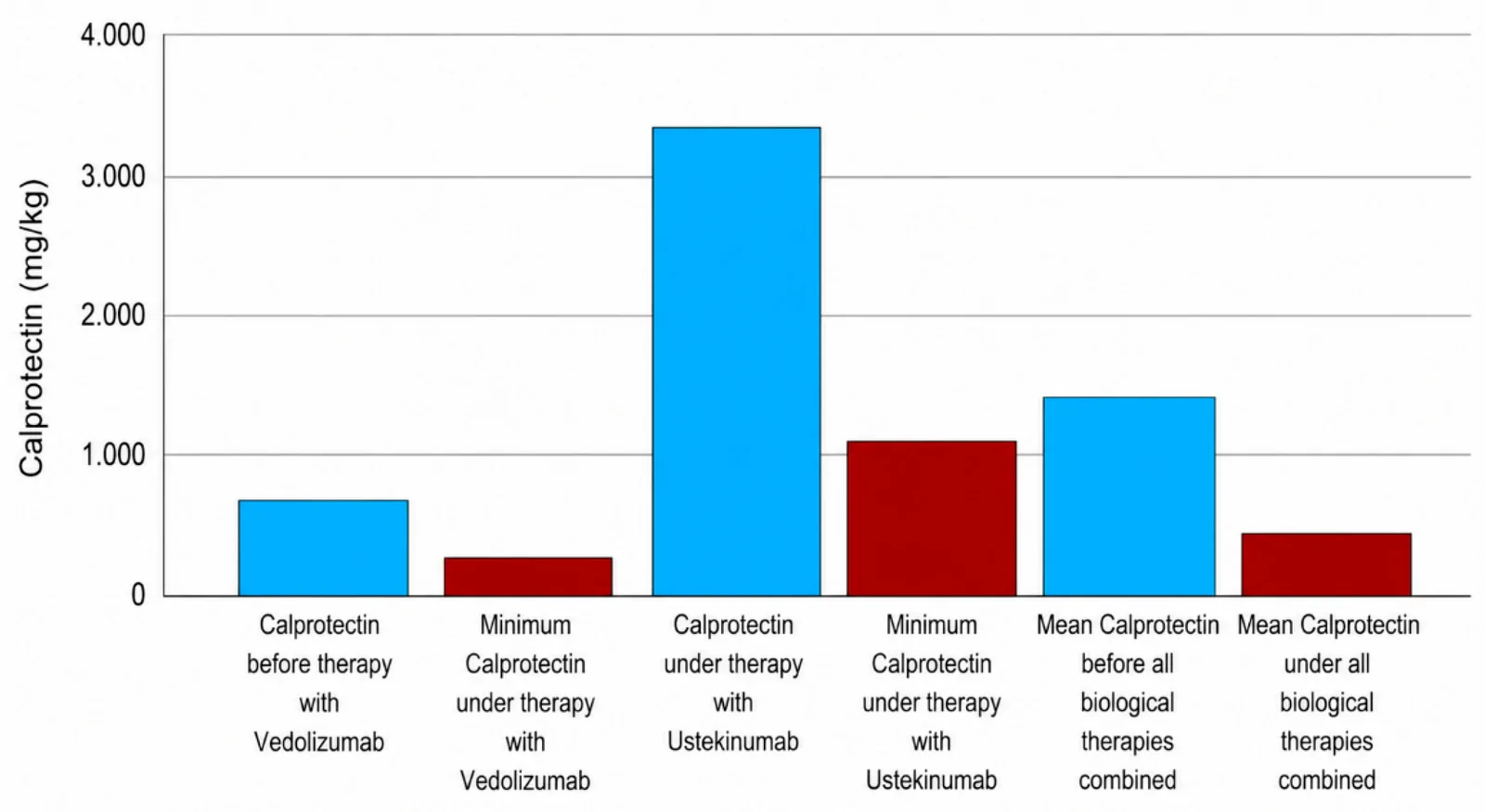
Calprotectin before and during biologic therapy.

Fecal calprotectin concentrations decreased during treatment with vedolizumab and ustekinumab (Figure 2). No data were available for patients treated with risankizumab or infliximab.

Mean fecal calprotectin levels decreased from 647 mg/kg to 188 mg/kg during vedolizumab therapy (70.2% reduction; *p* = 0.06) and from 3366 mg/kg to 1070 mg/kg during ustekinumab therapy (68.2% reduction; *p* = 0.25).

A similar pattern was observed for C-reactive protein (CRP). Median CRP levels decreased from 7.2 to 1.8 mg/L during vedolizumab therapy (75% reduction; *p* = 0.025) and from 10.0 to 3.5 mg/L during ustekinumab therapy (65% reduction; *p* = 0.004).

## 4. Discussion

Patients with IBD who undergo liver transplantation pose a particular therapeutic challenge: intestinal disease activity may persist or even flare despite maintenance immunosuppression, yet transplant recipients were systematically excluded from the pivotal trials of biologic agents, leaving the safety and effectiveness of combining biologics with post-transplant immunosuppression poorly defined. Our single-center analysis adds systematic real-world data to this sparse evidence base. In five liver transplant recipients requiring biologic therapy for IBD that remained active under maintenance immunosuppression, the combination of biologic agents with calcineurin inhibitors, mycophenolate mofetil, and corticosteroids proved feasible: we observed no acute graft rejection, no clinically apparent interactions, and no severe biologic-induced hepatotoxicity. Moreover, declining fecal calprotectin and CRP concentrations during vedolizumab and ustekinumab therapy indicate an objective biochemical response of intestinal inflammation. Taken together, these findings support biologic therapy as a good option for liver transplant recipients with active IBD, provided close clinical and laboratory monitoring is ensured.

Biochemical liver abnormalities were frequently observed during biologic therapy. Increases in γ-glutamyl transferase, AP, bilirubin, and GLDH were more pronounced than changes in aminotransferase levels. Importantly, these abnormalities were not observed immediately after initiation of a biologic agent and generally occurred in temporal proximity to active intestinal disease, biliary complications, recurrent cholangitis, or progression of the underlying graft disease. In several patients, alternative explanations were therefore clinically more plausible than biologic-induced liver injury. For example, anastomotic biliary strictures and recurrent PSC may produce a predominantly cholestatic biochemical pattern that is difficult to distinguish from potential drug-related injury in a retrospective analysis [6,11]. Moreover, the highest values observed during treatment with risankizumab were derived from a single patient and may therefore primarily reflect that individual’s clinical course rather than an agent-specific effect.

Drug-induced liver injury has been described with biologic agents, particularly anti-TNF therapies, but it remains uncommon and may manifest through several phenotypes, including hepatocellular injury and autoimmune hepatitis-like reactions [12]. In the present cohort, no consistent temporal pattern, progressive hepatocellular injury, or reproducible relationship between initiation of a biologic agent and deterioration of liver biochemistry was observed. The number of documented adverse events in our cohort was lower than that reported in the published systematic reviews. This finding should be interpreted cautiously. Adverse events were identified retrospectively from routine clinical documentation rather than through a predefined prospective surveillance protocol. Mild infections or events managed outside our institution may therefore have been missed.

Our findings concerning safety are consistent with the available literature. In the systematic review by Meyer et al., 187 solid organ transplant recipients receiving biologic therapy were identified, including 141 liver transplant recipients. IBD was the most common indication for biologic therapy, and anti-TNF agents and vedolizumab accounted for most exposures. Infections occurred in approximately 29% of patients, while malignancies and acute graft rejection were less frequent. However, the evidence was derived predominantly from individual case reports and small case series, with variable follow-up and likely substantial reporting bias [13]. A subsequent systematic review and meta-analysis specifically evaluating biologic and small-molecule therapy for IBD in solid organ transplant recipients included 163 patients, most of whom were liver transplant recipients. The pooled rates of all infections and serious infections were approximately 20 and 17 events per 100 person-years, respectively. No deaths attributable to biologic therapy were reported, and the authors concluded that biologic treatment appeared generally well tolerated, while emphasizing the need for longer-term and agent-specific data [14]. More recently, a dual-center retrospective study compared gut-selective vedolizumab with systemic biologics, including anti-TNF agents and ustekinumab, in 36 liver transplant recipients exposed during 59 biologic treatment episodes. This study did not identify a clear safety advantage of vedolizumab, suggesting that other agents may also be used after liver transplantation when clinically indicated [15].

Vedolizumab was selected as the first biologic agent in all our patients. This sequence had not been prospectively defined but reflected the clinical preference to initiate a gut-selective therapy with limited systemic immunosuppressive activity in patients already receiving post-transplant immunosuppression. In cases of insufficient response, treatment was subsequently changed to ustekinumab, risankizumab, or infliximab. The relatively uniform treatment sequence should therefore not be interpreted as evidence of superiority of one agent over another. It reflects the chronology of drug approval and availability, because several currently available IL-23-targeted therapies had not yet been approved or were not routinely accessible when the earlier treatment decisions were made. In our cohort, fecal calprotectin decreased by approximately 70% during both vedolizumab and ustekinumab therapy. In addition, CRP decreased significantly during both therapies.

At present, there is insufficient evidence to recommend a specific biologic agent for IBD in liver transplant recipients or for patients with PSC-associated IBD. Neither vedolizumab, ustekinumab, anti-TNF therapy, nor selective IL-23 inhibition has been shown to modify the course of PSC or recurrent PSC independently of its effect on intestinal inflammation. A recently reported case series of de novo IBD after solid organ transplantation described clinical and endoscopic responses to IL-23 pathway-targeted therapies without opportunistic infection or graft rejection, but the number of patients was small and the findings remain hypothesis-generating [16].

Control of intestinal inflammation may nevertheless be particularly relevant in patients transplanted for PSC. The relationship between post-transplant IBD activity and recurrent PSC has been inconsistent across studies. A large Nordic cohort found that colectomy before transplantation was associated with a lower risk of recurrent PSC, whereas IBD activity itself was not independently associated with recurrence [17]. In contrast, a more recent international multicenter study involving 531 liver transplant recipients identified increased post-transplant IBD activity as an independent risk factor for recurrent PSC [18]. A 2026 multicenter analysis also found concomitant IBD to be associated with an increased risk of PSC recurrence, although it did not establish that treatment of IBD prevents recurrence [19]. These apparently conflicting findings may reflect differences in the definition and measurement of IBD activity, the timing of endoscopic assessment, immunosuppressive regimens, and follow-up duration. Evidence regarding colectomy is similarly suggestive but not definitive. A systematic review found that several retrospective studies supported a protective association between pre- or peri-transplant colectomy and recurrent PSC, but the evidence was considered insufficient to justify colectomy solely for the prevention of PSC recurrence [20].

The biological plausibility of an interaction between intestinal and hepatobiliary inflammation is supported by the gut–liver axis. Alterations in intestinal barrier function, mucosal immunity, microbial composition, and enterohepatic trafficking of immune cells have all been implicated in PSC pathogenesis [7,21]. Microbiome studies in liver transplant recipients have demonstrated associations between mucosal inflammation and microbial composition, although a specific microbiome signature reliably predicting recurrent PSC has not yet been established [22]. Therefore, effective control of IBD after liver transplantation remains important for intestinal outcomes and may potentially influence graft outcomes, but it cannot currently be assumed that biologic therapy prevents recurrent PSC.

Several limitations must be considered, most of which are inherent to studying a rare population that has been systematically excluded from the pivotal trials of biologic agents. The retrospective single-center design limited the cohort to five biologic-exposed patients, but at the same time ensured uniform laboratory methodology and continuous follow-up. The heterogeneity of underlying liver diseases, transplant histories, IBD phenotypes, and biologic treatment sequences precludes agent-specific conclusions, yet mirrors the real-world setting in which these treatment decisions have to be made. As follow-up was not protocol-based and treatment exposure varied considerably, mild adverse events managed outside our center may have escaped documentation, although transplant-related care was largely centralized at our institution. These constraints reinforce the need for multicenter registries and prospective studies in this population.

Despite these limitations, the study provides detailed longitudinal information on a clinically challenging patient population. The inclusion of patients receiving vedolizumab, ustekinumab, risankizumab, and infliximab extends the available evidence beyond the predominantly anti-TNF- and vedolizumab-based literature. Our findings support the cautious use of biologic therapy in liver transplant recipients with active IBD when conventional treatment is insufficient, provided that patients undergo close interdisciplinary monitoring of infection, graft function, biliary complications, and intestinal disease activity.

In summary, there were no observed safety signals for biologic therapy in this cohort of liver transplant recipients with active IBD. Biologic therapy was associated with reductions in inflammatory biomarkers without a clear signal of clinically apparent drug-interactions, biologic-induced hepatotoxicity, severe infection, or graft-related complications.

## 5. Conclusions

In this retrospective single-center analysis, biologic therapy with vedolizumab, ustekinumab, risankizumab, or infliximab was feasible in liver transplant recipients with active IBD receiving maintenance immunosuppression: inflammatory biomarkers declined, and no signal of biologic-induced hepatotoxicity, clinically relevant drug–drug interactions, acute graft rejection, or severe infectious complications emerged.

Observed liver test abnormalities were more plausibly explained by disease-related factors than by biologic therapy, although causal attribution is not possible in a cohort of this size, and confirmation in prospective multicenter studies with standardized monitoring of graft function, infections, and IBD activity is warranted.

For clinical practice, our findings suggest that effective IBD therapy should not be withheld from liver transplant recipients for fear of combined immunosuppression: in carefully selected patients, biologics can be used under close interdisciplinary follow-up by gastroenterology, hepatology, and transplant medicine.

## Figures and Tables

**Table 1:** Patient characteristics.

	Total	Without Biologics	With Biologics
Number of patients; *n* (%)	17 (100.0)	12 (100.0)	5 (100.0)
Age; mean (range)	50.4 (34–67)	51.9 (36–67)	47.0 (34–65)
Sex; *n* (%)			
Female	5 (29.4)	3 (25.0)	2 (40.0)
Male	12 (70.6)	9 (75.0)	3 (60.0)
Underlying liver disease; *n* (%)			
AIH	4 (23.5)	1 (8.3)	3 (60.0)
PSC	11 (64.7)	10 (83.3)	1 (20.0)
PSC/AIH overlap syndrome	1 (5.9)	0 (0)	1 (20.0)
Cryptogenic cirrhosis	1 (5.9)	1 (8.3)	0 (0)
Underlying IBD; *n* (%)			
UC	14 (82.4)	10 (83.3)	4 (80.0)
CD	3 (17.6)	2 (16.7)	1 (20.0)
Number of liver transplantations; *n* (%)			
1	12 (70.6)	10 (83.3)	2 (40.0)
2	4 (23.5)	2 (16.7)	2 (40.0)
3	1 (5.9)	0 (0)	1 (20.0)
Maintenance immunosuppression after liver transplantation; *n* (%); multiple enumerations			
Tacrolimus	12 (70.6)	8 (66.7)	4 (80.0)
MMF	10 (58.8)	8 (66.7)	2 (40.0)
Steroids	9 (52.9)	5 (41.7)	4 (80.0)
Cyclosporin	4 (23.5)	3 (25.0)	1 (20.0)
Azathioprine	1 (5.9)	1 (8.3)	0 (0)
Sirolimus	1 (5.9)	1 (8.3)	0 (0)
Biologic therapy for IBD; *n* (%); multiple enumeration			
None	12 (70.6)	12 (100.0)	0 (0)
Vedolizumab	5 (29.4)	0 (0)	5 (100.0)
Ustekinumab	3 (17.6)	0 (0)	3 (60.0)
Risankizumab	1 (5.9)	0 (0)	1 (20.0)
Infliximab	1 (5.9)	0 (0)	1 (20.0)

**Table 2:** Number of patients and treatment months for each biologic agent.

Biologic Agent	Number of Patients	Number of Treatment Months
Vedolizumab	5	87
Ustekinumab	3	47
Risankizumab	1	5
Infliximab	1	3

**Table 3:** Adverse events during biologic therapy.

Biologic Therapy/Adverse Event	*n* (%)
Vedolizumab (*n* = 5)	
Uncomplicated infection (CTCAE Grade 1/2)	1 (20)
Complicated infection (CTCAE Grade 3/4)	1 (20)
Leukocytoclastic vasculitis (CTCAE Grade 2)	1 (20)
CMV colitis (CTCAE Grade 3)	1 (20)
Ustekinumab (*n* = 3)	
Hair loss (CTCAE Grade 1)	1 (33.3)
Risankizumab (*n* = 1)	0 (0)
Infliximab (*n* = 1)	0 (0)

## Data Availability

Original research data can be obtained on request.

## Abbreviations

The following abbreviations are used in this manuscript:

5-ASA	5-aminosalicylic acid
γGT	Gamma-glutamyltransferase
AIH	Autoimmune hepatitis
ALT	Alanine aminotransferase
AP	Alkaline phosphatase
AST	Aspartate aminotransferase
CBD	Common bile duct
CD	Crohn’s disease
CMV	Cytomegalie virus
CNI	Calcineurin inhibitors
CRP	C-reactive protein
ERCP	Endoscopic retrograde cholangiopancreatography
GLDH	Glutamate dehydrogenase
IBD	Inflammatory bowel disease
IL	Interleukin
LT	Liver transplantation
MMF	Mycophenolate mofetil
MRCP	Magnetic resonance cholangiopancreatography
MRI	Magnetic resonance imaging
MTX	Methotrexate
PSC	Primary sclerosing cholangitis
PTCD	Percutaneous transhepatic cholangial drainage
TNF	Tumor necrosis factor alpha
UC	Ulcerative colitis
ULN	Upper limit of normal
